# Dynamic membrane filtration accelerates electroactive biofilms in bioelectrochemical systems

**DOI:** 10.1016/j.ese.2023.100375

**Published:** 2023-12-27

**Authors:** Jinning Wang, Mei Chen, Jiayao Zhang, Xinyi Sun, Nan Li, Xin Wang

**Affiliations:** a MOE Key Laboratory of Pollution Processes and Environmental Criteria, Tianjin Key Laboratory of Environmental Remediation and Pollution Control, College of Environmental Science and Engineering, Nankai University, No. 38 Tongyan Road, Jinnan District, Tianjin, 300350, China; b School of Environmental Science and Engineering, Tianjin University, No. 35 Yaguan Road, Jinnan District, Tianjin, 300350, China

**Keywords:** Electroactive biofilm, Membrane filtration, Spatial structure, Mass transfer, Microbial community

## Abstract

Bioelectrochemical systems (BES) have emerged as a dual-function technology for treating wastewater and recovering energy. A vital element of BES is the rapid formation and maintenance of electroactive biofilms (EABs). Previous attempts to accelerate EAB formation and improve electroactivities focused on enhancing the bacterial adhesion process while neglecting the rate-limiting step of the bacterial transport process. Here, we introduce membrane filtration into BES, establishing a dynamic membrane filtration system that enhances overall performance. We observed that optimal membrane flux considerably reduced the startup time for EAB formation. Specifically, EABs established under a 25 L m^−2^ h^−1^ flux (EAB_25 LMH_) had a formation time of 43.8 ± 1.3 h, notably faster than the 51.4 ± 1.6 h in the static state (EAB_0 LMH_). Additionally, EAB_25 LMH_ exhibited a significant increase in maximum current density, approximately 2.2 times higher than EAB_0 LMH_. Pearson correlation analysis indicated a positive relationship between current densities and biomass quantities and an inverse correlation with startup time. Microbial analysis revealed two critical findings: (i) variations in maximum current densities across different filtration conditions were associated with redox-active substances and biomass accumulation, and (ii) the incorporation of a filtration process in EAB formation enhanced the proportion of viable cells and encouraged a more diverse range of electroactive bacteria. Moreover, the novel electroactive membrane demonstrated sustained current production and effective solid-liquid separation during prolonged operation, indicating its potential as a viable alternative in membrane-based systems. This approach not only provides a new operational model for BES but also holds promise for expanding its application in future wastewater treatment solutions.

## Highlights

• Current density of EABs under filtration were 2.2 times higher than conventional EAB.
• Filtration process increased microbial production and modulated their compositions.
• Biomass amount played vital roles in improving electrochemical performance.
• EABs under filtration kept excellent current output during long-term operation.
• This research provides a novel strategy for enhancing overall performance of EAB.

## Graphical abstract

**Figure:**
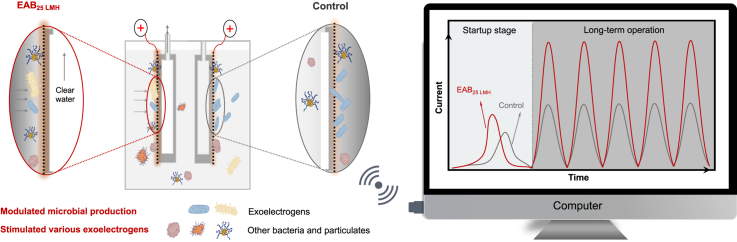

## 1 Introduction

Bioelectrochemical systems (BES) have shown considerable promise in wastewater treatment and energy recovery owing to their unique abilities, including electrical current generation, valuable chemical production, and contaminant remediation [[1], [2], [3]]. In BES, the electrochemically active biofilms (EABs) play crucial roles in degrading organic matters, transferring electrons, and generating electrical energy [4], regarded as the most vital element in determining the extracellular electron transfer ability and the BES efficiency [5]. Therefore, quickly forming EABs with high electrochemical properties and maintaining sustained current output during long-term operation is imperative for the high efficiency and durable operation of BES.

Various attempts have been made to accelerate EAB formation and establish EABs with higher electroactivities. These attempts included anodic surface modification [6,7], mediator introduction [8], and optimization of operational conditions (e.g., applied voltage, pH, substrate concentration, etc.) [[9], [10], [11]]. For instance, nanocoating materials have been proven to promote EAB growth by increasing the electrode-specific surface area and enhancing electrical conductivity and extracellular electron transport [12]. Studies have also demonstrated that electrodes functioning with hydrophilic, charged groups could enhance the biofilm attachment, thus improving BES performance [6]. Notably, most recent studies mainly focused on strengthening the long-range/short-range force (e.g., electrostatic interactions, hydrophilic interactions, van der Waals interactions, hydrogen bonding, etc.) between bacteria and electrodes to enhance the bacterial adhesion process [[6], [7], [8]]. However, they neglected to expedite the bacterial transport process [13]. It should be noted that bacterial transport onto the surface is a possible rate-limiting step for biofilm formation [13]. Furthermore, these strategies are ineffective in alleviating diffusive constraints and proton accumulation in EABs, which are the main reasons for the active cell decay and reduced current generation [14,15].

Membrane filtration technologies have prominent advantages, including efficient separation, high effluent quality, and low footprint requirements [16,17]. To take advantage of both BES and membrane filtration, their integration was investigated to achieve high-quality effluent, the potential of membrane fouling mitigation, and energy recovery. For instance, Katuri et al. [18] and Werner et al. [19] combined microbial electrolysis cells with a membrane bioreactor to mitigate membrane fouling due to the *in-situ* hydrogen production on the cathodic membrane surface. Our previous research also found that EABs formed on cheap, coarse, and conductive substrates by electrical stimulation could serve as dynamic membranes and biocatalysts for efficient separation and contamination removal [20]. Although such research provides vital insights into integrating membrane filtration and BESs as next-generation energy-efficient wastewater technology [21], the roles of membrane filtration on BES performances, especially on EAB formation, are overlooked. Since the enhanced mass transfer rate of target matters to filter surface has been proven in membrane filtration systems under flow-through mode [22,23], we hypothesized that the increased mass transfer and efficient solid-liquid separation of the filtration process might facilitate the transport and adhesion process to facilitate EAB formation. Moreover, the convection transport of water increased by the filtration process has been reported to decrease the diffusion boundary layer [24]. Notably, this may be conducive to addressing substrate limitation and proton accumulation, thus sustaining EABs metabolically.

Therefore, we developed an electroactive membrane filtration system using carbon cloth as an anode and filter substrate to enhance BES performance. As such, the filtration impacts on the startup time (*t*_start-up_), current output, and electrochemical properties were investigated. Furthermore, batch tests were carried out to explore the roles of interception and mass transfer enhancement on electroactivity improvement. EABs formed under different fluxes were further systematically analyzed to reveal the mechanism of electroactivity improvement.

## 2 Material and methods

### 2.1 Inoculation

Microbial suspension from microbial fuel cells and domestic wastewater from wastewater treatment plants in the Jinnan campus of Nankai University were mixed at a ratio of 1:11 and used as the inoculum. The medium containing 1 g L^−1^ NaAc, 12.5 mL L^−1^ trace minerals (details in Supporting Information (SI) Table S1), 5 mL L^−1^ vitamin solution (Table S2) and 50 mM phosphate buffer (PBS: 4.58 g L^−1^ Na_2_HPO_4_; 2.13 g L^−1^ NaH_2_PO_4_; 0.31 g L^−1^ NH_4_Cl; 0.13 g L^−1^ KCl) was used to cultivate microorganisms [25]. Before use, the medium was sparged with N_2_/CO_2_ gas (4:1 [vol/vol]) for 20 min to eliminate dissolved oxygen.

### 2.2 Experimental set-up and operation

#### 2.2.1 Fabrication of electrode module

Carbon cloth (77% in porosity, 5 cm × 5 cm in size) (CeTech Co., Ltd, China) with high specific surface area, good hydrophilic properties, and good electrical conductivity was used as the anodic filtration substrate [20]. Prior to use, the carbon cloth was washed with alcohol, diluted with hydrochloric acid followed by nitric acid, and then rinsed with deionized water. To fabricate an electrochemical filtration module with robustness, a titanium (Ti) mesh (ChaoChuang Metal Mesh Co., Ltd, Hebei Province, China) with pore size of 0.8 mm × 1.2 mm and thickness of 0.1 mm was used as the current collector and filtration support material for providing mechanical strength. In brief, the Ti mesh and the carbon cloth were orderly covered on an acrylic bracket using epoxy resin adhesive, according to Fig. S1a. Additionally, Ti wire (FuXiang Material Co., Ltd, Hebei Province, China) was used to connect the working electrode of the electrochemical filtration module to a multi-channel potentiostat (DH7006, Donghua Instrument, Jiangsu, China).

#### 2.2.2 Reactor configuration and operation

Unless otherwise stated, all experiments were carried out in a microbial electrolysis cell reactor with a volume of 2000 mL (Fig. S1b). Considering the possible applied scenario of integrating BES and membrane filtration, the applied membrane fluxes in anaerobic membrane bioreactors (5–15 L m^−2^ h^−1^) were referred [26,27]. Moreover, since high filtration flux is one distinctive advantage of dynamic membranes compared to the commonly used microfiltration and ultrafiltration membranes [28], three groups of electrodes were placed in the reactor in parallel. Different membrane fluxes (0, 25, and 50 L m^−2^ h^−1^) from extracting medium from the fabricated modules were conducted to regulate the formation of different EABs (EAB_0 LMH_, EAB_25 LMH,_ and EAB_50 LMH_, respectively). The fabricated modules were used as the working electrode, while a platinum sheet (5 cm × 5 cm in size) and an Ag/AgCl electrode (4 M KCl, 0.197 V versus the standard hydrogen electrode, SHE) were used as the counter and reference electrodes in each group. The cathode and anode of each group were fixed in the slots at a 2 cm distance. Additionally, the multi-channel potentiostat was employed to provide a poised potential of 0 V (vs. Ag/AgCl) to develop EABs, and chronoamperometry (CA) was used to record the current every 100 s. Electrochemical tests, including turnover cyclic voltammetry (CV), nonturnover CV, the first derivative CVs (DCVs), and electrochemical impedance spectroscopy (EIS), were carried out (detailed methods could be found in Section S1) at representative times (e.g., current density arrived the highest). Three reactors, as mentioned, were operated in parallel to investigate the influences of filtration on EAB start-up. Following the start-up cycle, EABs in two reactors were used to analyze the properties of different EABs. The last one was kept operating to examine the long-term stability of EAB under filtration conditions. During the long-term operation, the medium in the reactor was changed once the current density was smaller than 0.05 A m^−2^ during long-term experiments.

Batch tests were performed to elucidate the contributions of interception and the mass transfer enhancement induced by filtration on EAB formation. Briefly, the fabricated modules were pre-filtrated with the mixed inoculum under different fluxes (0, 25, and 50 L m^−2^ h^−1^) at predetermined times to attach different amounts of microorganisms physically. The predetermined time was set as 24 h according to the growth curve of most microbes [29], which was also enough for circulating all medium in the reactor once. The initial biomass of EAB after pre-filtrated different in 0, 25 and 50 L m^−2^ h^−1^ was 2.3 ± 0.4, 12.0 ± 1.1, 22.1 ± 0.3 μg cm^−2^, respectively. After switching the solution to the cultivated medium without microorganisms, pre-filtrated modules with different amounts of microorganisms were connected to the potentiostat and cultivated at a static state. In contrast, the electrode modules were circulated at the same flux to attach uniform microorganisms. Subsequently, the pre-filtrated modules were connected to the potentiostat and operated at different fluxes (0, 25, and 50 L m^−2^ h^−1^) after refreshing the medium.

### 2.3 Analytical methods

#### 2.3.1 Micromorphology observation and fluorescence *in situ* hybridization

Confocal laser scanning microscopy (CLSM) (LSM880, Zeiss, Germany) was performed to observe the spatial topography of EABs following the first cycle. The biofilms were stained with a LIVE/DEAD BacLight bacterial viability kit (Invitrogen, CA) for 20 min and then rinsed with 50 mM PBS twice before imaging. The thickness of the biofilms was measured via CLSM under Z-stack mode with a scanning step of 2 μm. Fluorescence *in situ* hybridization (FISH) was used to further detect the distribution of *Geobacter* spp. in biofilms. Geo825-FITC (TACCCGCRACACCTAGT) was used as *a Geobacter-*specific probe to target *Geobacter* cells, and DAPI Nucleic Acid Stain (cat. No. D1306, Thermo Fisher Scientific, Waltham, MA) was used to stain all bacteria in the biofilms. The labeled EABs were imaged visually by CLSM via Z-stack mode (10× object, 2 μm scanning step). Image J's three-dimensional (3D) viewer plugin was applied to reconstruct the 3D biofilm images. Furthermore, field emission scanning electron microscopy (FESEM) (Model: JEOL JSM 7800F) was conducted to observe the surface morphology of EABs (Section S2).

#### 2.3.2 Microbial community analysis

Biological samples on EABs were collected by scraping the electrode surface using sterile blades. Genomic DNA was extracted using the Soil Genomic DNA Kit (CW2091S, ComWin Biotech Co., Ltd., Beijing, China). Based on the hypervariable V4 region of the 16S rRNA gene, a primer set of 515F (GTGCCAGCMGCCGCGGTAA) and 806R (GGACTACHVGGGTWTCTAAT) were selected to amplify the genes. Moreover, sequencing libraries were prepared and sequenced using the Illumina Miseq platform with the PE250 sequencing strategy (Shanghai Majorbio Bio-pharm Technology Co., Ltd, Shanghai, China).

#### 2.3.3 Other items

The biomass content and extracellular polymeric substance (EPS) were extracted and measured according to Yan et al. [30] (Section S3). According to a previous investigation, Cytochrome C, a relevant electron transfer enzyme, was extracted and detected based on the electrode surface area [31]. The concentration of cytochrome C was measured using a visible ultraviolet spectrophotometer. Furthermore, an analysis of variance (ANOVA) was carried out to evaluate the significant difference between the results, and *p* < 0.05 was considered statistically significant.

## 3 Results and discussion

### 3.1 Electrochemical properties of EABs under various membrane fluxes

**Fig. 1:**
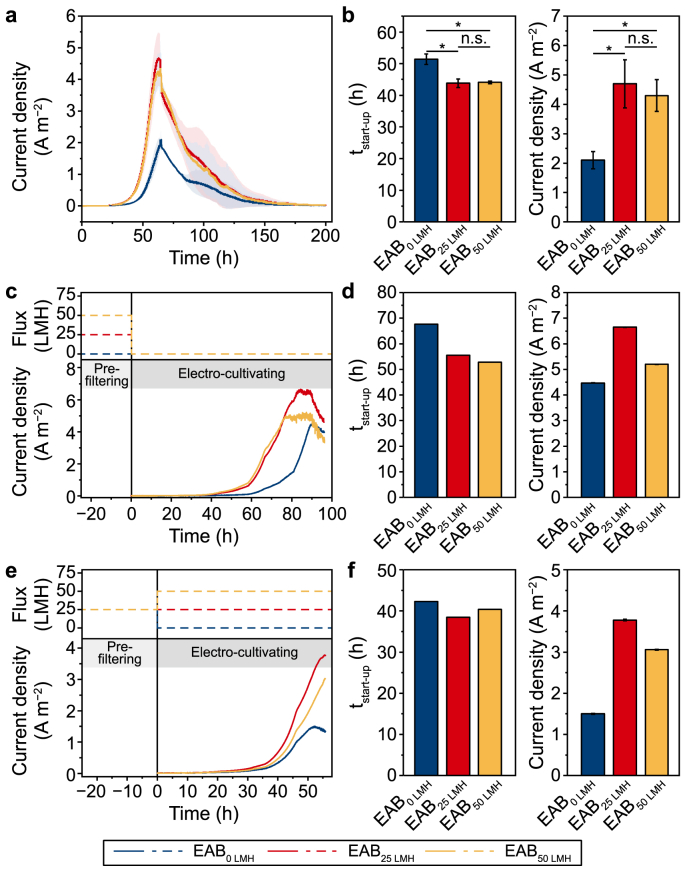
Performance of EABs under different fluxes conditions. **a**, Current density over time of EABs under different fluxes. **b**, *t*_start-up_ and maximum current density of three EABs (*n* = 3). **c**–**d**, Time-current curves (**c**), *t*_start-up_, and maximum current density (**d**) of different EABs in batch tests with pre-filtrated under different fluxes and electro-cultivated at static state. **e**–**f**, Time-current curves (**e**), *t*_start-up_, and maximum current density (**f**) of different EABs in batch tests with pre-filtrated under the same flux and electro-cultivated under different fluxes. (^n.s*.*^*p* > 0.05, ∗*p* < 0.05). In panels **c** and **e**, the dashed lines represent the flux conditions during the pre-filtering and electro-cultivation processes, and the solid lines represent the current generation during the electro-cultivating process.

The current production curves of all EABs in this study (Fig. 1a) exhibited the representative “S” shape with lag phase, log phase, and decline due to nutrient depletion, consistent with the typical biofilm growth [32]. Previous studies indicated that the current generation of EABs linearly correlated with the biofilm thickness and the increase of exoelectrogens colonization [6,33]. Therefore, the time taken to the point when current density approached 0.50 A m^−2^, reported as biofilms transition from monolayer to multilayers [34], was defined as the *t*_start-up_ to evaluate the biofilm formation process. The *t*_start-up_ of EABs under filtration (EAB_25 LMH_ and EAB_50 LMH_) was around 44 h, shorter than that of EAB_0 LMH_ formed at a static state (51.4 ± 1.6 h). This phenomenon is likely ascribed to the mass transfer improvement and the enhanced accumulation of microbes on an electrode by introducing filtration [35]. Bacterial adhesion on the electrode surface usually occurs after bacteria transport onto the surface, and the adhesion process would be influenced by physical-chemical forces (e.g., van der Waals, hydrophilic interactions, electrostatic interaction, etc.) [13]. Filtration introduced in EAB formation could likely facilitate bacteria transport to the electrode and strengthen the bacterial adhesion on the porous electrode by pore clogging. Furthermore, the surface morphology images of three EABs (Fig. S2) demonstrated that the surface of electrode modules under filtration conditions was covered with much denser microorganisms.

The maximum current density in EAB_25 LMH_ and EAB_50 LMH_ were significantly enhanced. The EAB_0 LMH_ control group exhibited the lowest maximum current (2.1 ± 0.3 A m^−2^), 55.3% lower than EAB_25 LMH_. Statistical analysis demonstrated that the performances (including *t*_start-up_ and the maximum current) of EAB_25 LMH_ and EAB_50 LMH_ were not significantly different. However, they were significantly different from the control group (EAB_0 LMH_) (Fig. 1b). These results demonstrated that the presence of filtration on EAB formation could shorten the startup time and enhance the biofilm electroactivity. Notably, the *t*_start-up_ and the maximum current of EAB_25 LMH_ were slightly better than that of EAB_50 LMH_, implying that the excessive flux may induce side effects by intensifying the hydraulic shear force and shortening the hydraulic retention time of the substrate [22].

Different performances of these EABs might be ascribed to the differences either in biomass amount, in mass transfer, in extracellular electron transfer rate, or in the microbial community. Batch experiments of pre-filtering different amounts of microorganisms on electrode modules followed by static electro-cultivating conditions and electro-cultivating the same initial biomass under different filtration conditions were performed to distinguish the contributions of biomass amount and mass transfer enhancement. As can be seen from Fig. 1c and d, the *t*_start-up_ of the EAB pre-filtrated under 50 L m^−2^ h^−1^ was the shortest (52.8 ± 0.1 h), shorter than the EAB under 0 L m^−2^ h^−1^ (67.7 ± 0.2 h). This result suggested that the more accumulated microorganisms on the electrode module would facilitate exoelectrogen colonization and decrease the EABs *t*_start-up_, indicating the crucial role of separation by filtration on EABs formation. However, the maximum current density of the EAB pre-filtrated under 25 L m^−2^ h^−1^ (6.7 ± 0.2 A m^−2^) was the highest, even much higher than the EAB pre-filtrated under 50 L m^−2^ h^−1^ (5.2 ± 0.1 A m^−2^). The phenomenon may be ascribed to the more compact biofilm formed under 50 L m^−2^ h^−1^, leading to more severe mass transfer limitations under static conditions. The *t*_start-up_ result of EABs covered with the same microorganisms and then cultivated under different fluxes (Fig. 1e and f) exhibited no significant difference, demonstrating that the accumulated biomass played a vital role in EAB formation. However, the much higher current density of the EABs under filtration (Fig. 1f) revealed that an advection-enhanced transport of substrates toward the biofilm could improve EAB electroactivity. Pearson correlation analysis indicated that the maximum current densities of EABs were positively correlated with the accumulated biomass amounts (*r* = 0.669) and mass transfer rate (*r* = 0.329). Moreover, the *t*_start-up_ of EABs had negative correlations with biomass amounts (*r* = −0.938). In summary, dual functions of interception and mass transfer enhancement by filtration are conducive to improving EAB electroactivity, and solid-liquid filtration separation promotes the adhesion of bacteria, thus accelerating the formation of electroactive biofilm.

**Fig. 2:**
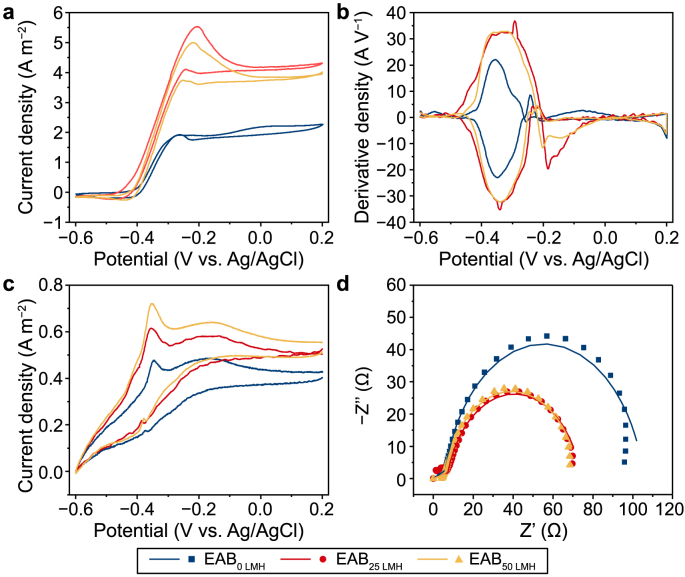
Electrochemical properties of three EABs at the first cycle stage. **a**, Turnover cyclic voltammetry. **b**, Derivate cyclic voltammetry. **c**, Nonturnover cyclic voltammetry. **d**, Electrochemical impedance spectroscopy.

Cyclic voltammetry (CV) was conducted to evaluate the electron transfer performance of EABs formed under different fluxes. According to the CV curves (Fig. 2a), the maximum current density of EAB_25 LMH_ (4.3 A m^−2^) and EAB_50 LMH_ (4.0 A m^−2^) was comparable and about twice higher than that of EAB _0 LMH_ (2.2 A m^−2^). This result indicated that EABs formed under filtration conditions equipped with more redox active components and exhibited better redox activity than the control of EAB_0 LMH_ [36]. A dominant pair of symmetrical peaks centered at −0.33 ± 0.03 V (vs. Ag/AgCl) were observed in the DCV of EABs (Fig. 2b), implying that the main electroactive substances (e.g., cytochrome) of these EABs are the same (duplicated result in Fig. S3). Following starvation in an acetate-lacking medium, a non-turnover CV of EABs was performed to further evaluate the kinetics of reversible redox-active proteins in biofilms [37]. All EABs exhibited a typical double peak voltammetric signature [38]. Regarding peak positions, the oxidative peaks at −0.35 V and −0.16 V and the reductive peaks at −0.37 V were present in all EABs non-turnover CV curves. Similar current responses were observed from the CV scans under non-turnover conditions. The redox peak height of biofilms followed the order EAB_25 LMH_ > EAB_50 LMH_ > EAB_0 LMH_, further confirming the better electroactive performance of EAB_25 LMH_ (Fig. 2c). Furthermore, the inflexion points of the CV of EABs were nicely agreed with the reported formal potentials of cytochrome C [39,40], which were also detected in all EABs in this study and had correlation with electroactivity. Thus, we hypothesized that cytochrome C might be the main redox active component, and the filter condition adjusted the content of the redox active components. These results indicated that EABs formed under different filtered conditions might have a similar electron transfer process. Namely, filtration promoted EABs electroactivity by increasing the amount of redox-active components but was not affected by the kinetics of the redox reactions [6].

### 3.2 Microbial products in EABs

**Fig. 3:**
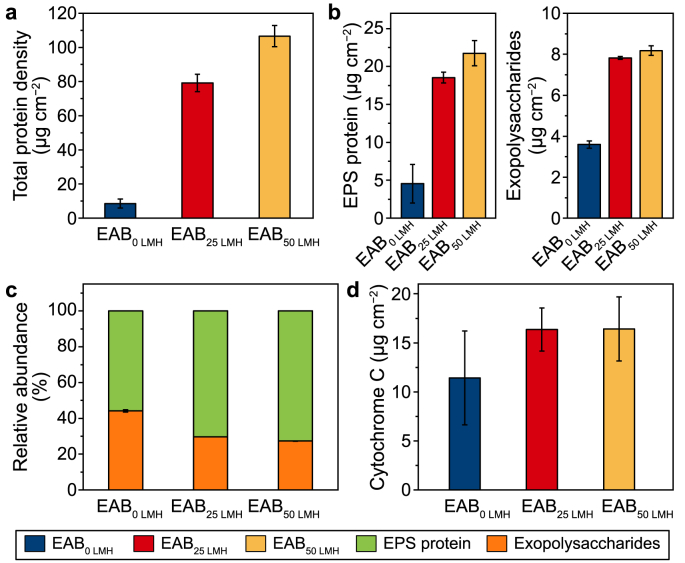
The response of EABs microbial products and cytochrome C to the variation of membrane fluxes after the first cycle. **a**, Total protein density. **b**, Proteins and exopolysaccharides in extracted EPS. **c**, The relative abundance of protein and polysaccharides in EPS. **d**, Cytochrome C.

The biofilm biomass of EABs was evaluated based on the total protein content after the first cycle [37]. As can be seen from Fig. 3a, the biomass amount in EABs increased with membrane flux increasing, which were 8.6 ± 2.7, 79.2 ± 5.1, and 106.6 ± 6.2 μg cm^−2^ for EAB_0 LMH_, EAB_25 LMH_ and EAB_50 LMH_, respectively. The high biomass in EAB with filtration might positively enhance electroactivity by increasing the absolute amount of exoelectrogens [41]. Microbial products of EPS have been reported to protect cells against environmental stress [42] and as a storage of electroactive components (e.g., cytochrome C) to transport extracellular electrons [43]. In this regard, the main components of protein and polysaccharides in EPS were analyzed, and the values varied among the EABs formed under different fluxes (Fig. 3b). Both the contents of EPS-protein and exopolysaccharides in EABs increased with fluxes increased from 0 LMH to 50 LMH. Compared to EAB_0 LMH_ (4.5 ± 2.5 μg cm^−2^), the significant increase of EPS-protein in EAB_25 LMH_ (18.5 ± 0.7 μg cm^−2^) and EAB_50 LMH_ (21.8 ± 1.7 μg cm^−2^) were responsible for the enhanced electroactivity, since EPS-protein contained various redox molecules to overcome the insulativity of EABs (mainly polysaccharides) [44,45] (Fig. 3c).

Cytochrome C is vital in the extracellular electron transfer process as the membrane-bound redox protein [46]. Thus, the cytochrome C content was further explored to investigate the electroactivity of EABs. Similar cytochrome C contents were observed in EAB_25 LMH_ (16.3 ± 2.2 μg cm^−2^) and EAB_50 LMH_ (16.4 ± 3.2 μg cm^−2^). In comparison, EAB_0 LMH_ (11.4 ± 4.8 μg cm^−2^) had the lowest level, consistent with the better electrochemical activity of EAB_25 LMH_ and EAB_50 LMH_ (Fig. 3d).

### 3.3 Spatial structure of EABs

**Fig. 4:**
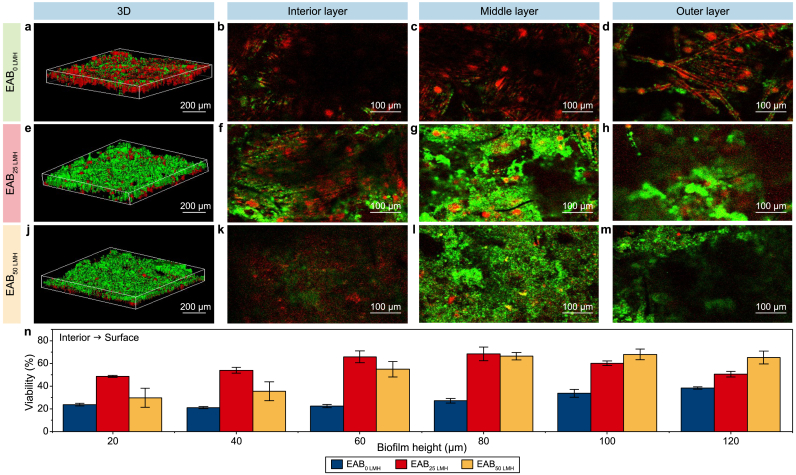
**a**–**m**, Spatial distribution images of the live cell (green color) and dead cell (red color) in EAB_0 LMH_ (**a**–**d**), EAB_25 LMH_ (**e**–**h**), and EAB_50 LMH_ (**j**–**m**) by CLSM analysis, including the three-dimensional image (**a**, **e**, **j**), the interior layer near the electrode (**b**, **f**, **k**), the middle layer (**c**, **g**, **l**) and the outer layer (**d**, **h**, **m**) face to the solution. **n**, Variation of viability in EABs with thickness.

The spatial structure and viabilities of EABs under different membrane fluxes were investigated with CLSM imaging since the viable cells of EAB play vital roles in electron production. As shown in Fig. 4, the distribution of viable cells in all EABs was heterogeneous, and the introduction of the filtration process visibly changed the internal structure of EABs. A higher fluorescence intensity and viability in the interior, middle, and outer layers were observed in EAB_25 LMH_ and EAB_50 LMH_ than in EAB_0 LMH_, which coincides with the surface morphology images of EABs (Fig. S2). Furthermore, a two-layer structure (a live outer layer that covered a dead inner core) was observed in the control of EAB_0 LMH_ due to the accumulation of proton [47] and the limiting condition of electron donors [48]. It should be noted that the viability of EABs formed under filtration exhibited more heterogeneity. In comparison, the much higher viable cells were presented in the outer biofilm layer of EAB_0 LMH_ (23.7 ± 1.1%), while the maximum viabilities of both EAB_25 LMH_ (68.5 ± 6.0%) and EAB_50 LMH_ (68.0 ± 5.6%) were observed in the middle layer. This phenomenon should be attributed to the fact that the advection-enhanced transport induced by introducing filtration would alter the transfer of substrate and proton [35]. Krieg et al. [49] also found that an optimized substrate distribution by altering the flow pattern could increase microbial fuel cell performance.

**Fig. 5:**
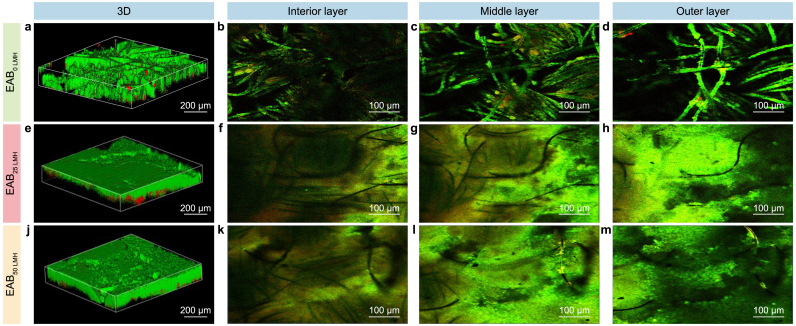
Fish images of *Geobacter* spp. in EAB_0 LMH_ (**a**–**d**), EAB_25 LMH_ (**e**–**h**), and EAB_50 LMH_ (**j**–**m**), including the three-dimensional image (**a**, **e**, **j**), the interior layer near the electrode (**b**, **f**, **k**), the middle layer (**c**, **g**, **l**) and the outer layer (**d**, **h**, **m**) face to the solution. *Geobacter* spp. were imaged as red, whereas all cells were imaged here as green.

FISH analysis was carried out to qualitatively observe the distribution of the *Geobacter* genus (red) and all bacteria (green). Fig. 5 presents the 3D image and internal layers of EABs formed under various fluxes. Layer-by-layer images of FISH (Fig. 5a–c) show that most *Geobacter* in EAB_25 LMH_ and EAB_50 LMH_ are mainly distributed in the interior and middle layers, slightly different from the control in static conditions. The enhanced mass transfer and convenient electron transport might contribute to regulating the distributions of exoelectrogens (e.g., *Geobacter*) in EAB under filtration. The surface coverage and biomass amount on three EABs were presented in Table S3. Biomass covered 62.7% and 80.3% of EAB_25 LMH_ and EAB_50 LMH_ electrode surfaces but only around 19.3% of the EAB_0 LMH_ surface. Notably, the enhanced transport and adhesion of bacteria toward electrodes should be responsible for the higher biomass coverage of EAB_25 LMH_ and EAB_50 LMH_. The higher biomass quantity, which might lead to higher exoelectrogen content, could be the main reason for the enhanced electroactivity [6]. The biomass amount calculated based on biofilm coverage and thickness data also exhibited a similar tendency, consistent with the total protein result.

### 3.4 Analysis of microbial communities

High-throughput sequencing technology was performed to investigate the impacts of various fluxes on microbial communities. The results of the α-diversity index are presented in Table S4. Based on the coverage index, the EAB samples attained 0.99, demonstrating that the sequence analysis could nearly cover the bacterial population and the equivalent number of operational units (OTUs). The Sobs and Shannon indexes of the three EABs were lower than those of the inoculum, possibly due to the medium's homogeneity [50].

**Fig. 6:**
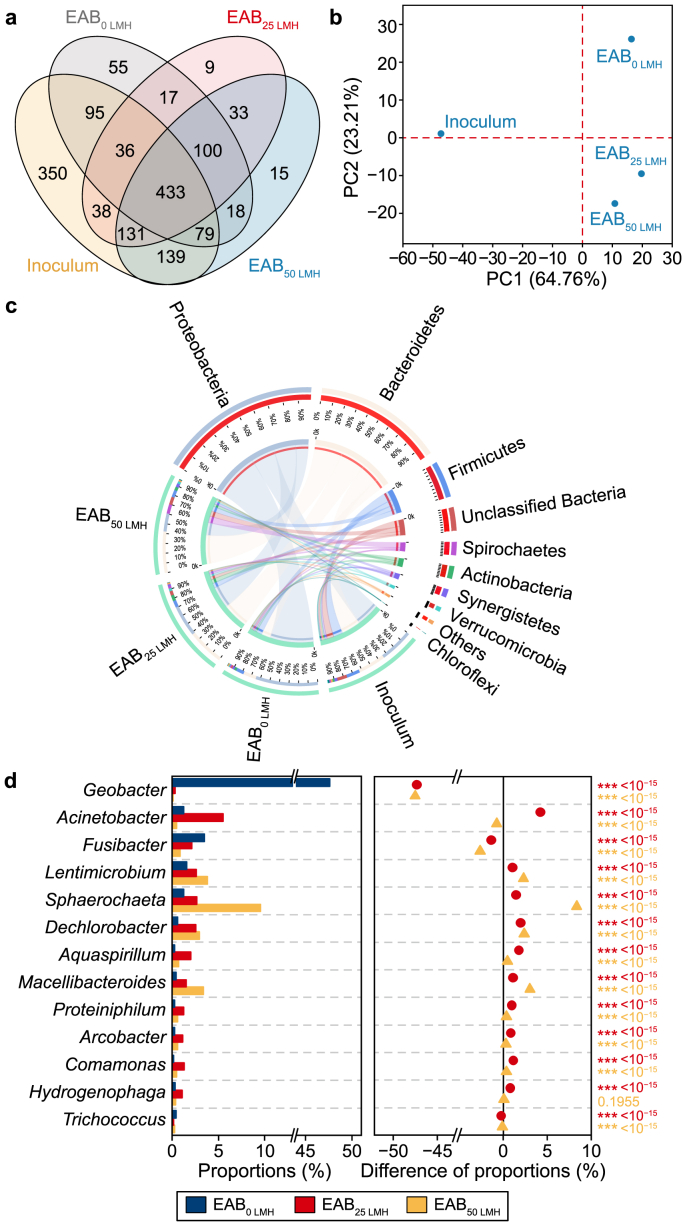
**a**–**b**, Venn graph (**a**) and principal component analysis (**b**) of microbial communities based on OTUs level. **c**–**d**, Microbial community distribution at the phylum level (**c**) and variations of the predominant phylogenetic genera (**d**) in three EABs. In panel **d**, the circles represent the statistical differences between EAB_25 LMH_ and EAB_0 LMH_, while the triangles indicate the differences between EAB_50 LM_ and EAB_0 LMH_.

Venn diagram analysis was performed to distinguish the similarities and differences based on the OTU level. The total number of observed OTUs in all samples was 3879, while only 1.1% of the total OTUs (433 OTUs) were shared, implying that most species in these samples differed. Notably, the shared OTUs between Inoculum and EAB_50 LMH_ was 782, accounting for 82.5% of the OTUs in EAB_50 LMH_. This result indicated that introducing a filtration process during EAB formation might keep the bacteria homology by accelerating transport and enhancing the initial adhesion of microflora in the inoculum to the electrode surface. Moreover, the shared OTUs in EAB_25 LMH_ and EAB_50 LMH_ was 697, which occupied 87.5% of the OTUs in EAB_25 LMH_, implying that the filtration process exhibited a selective role in microbial succession. Principal component analysis based on OTUs was used to further evaluate the distance and variations among all samples (Fig. 6b). PC 1 and PC 2 revealed 64.8% and 23.2% of the entire community variations, respectively. Moreover, EAB_25 LMH_ and EAB_50 LMH_ samples clustered closely together and far away from the Inoculum and EAB_0 LMH_ samples, which is consistent with the Venn analysis.

The predominant taxa were analyzed to compare the microbial structure in all samples at the phylum level. As shown in Fig. 6c, Proteobacteria, Bacteroidota, and Firmicutes were the three most abundant phyla. As the predominant abundant phyla, the total relative abundance of Bacteroidota and Proteobacteria exceeded 65% in each EAB sample. According to previous literature [51,52], Proteobacteria contained various confirmed exoelectrogens, and Bacteroidetes is also regarded as a putative phylum of exoelectrogens in bioelectrochemical systems.

A pairwise statistical analysis revealed the microbial community's function (Fig. 6d). Geobacter, the representative exoelectrogens, accounted for 48% of EAB_0 LMH_. Interestingly, the abundance of *Geobacter* in EAB_25 LMH_ (0.3%) and EAB_50 LMH_ (0.1%) exhibited insignificant improvement compared with the inoculum (0.1%). Notably, the abundance of *Geobacter* in the control group (48%), cultured in static conditions like traditional EAB was consistent with previous studies conducted in a phosphate buffer medium (50 mM) containing acetate as the only carbon source [53]. Intriguingly, the abundance of *Geobacter* under filtered conditions was significantly lower. This phenomenon is due to the increased absolute biomass under filtered conditions and the circulation flow mode. On the one hand, filtration enhanced the transport of various microorganisms in inoculum toward the electrode, which the Venn diagram analysis could prove. On the other hand, except for the electrostatic adsorption process of electrogenic bacteria in traditional EAB, the microorganisms in EABs under filtration conditions also derived from filtering of suspended communities, which normally consisted of little *Geobacter* based on the available literature [53]. This phenomenon indicated that electrical stimulation plays a main role in conventional EAB formation and preferentially screens exoelectrogens due to the electrotaxis. In contrast, introducing the filtration process provides another crucial impetus for microbial transport and adhesion [20]. The results further demonstrated that the relative abundance of various putative exoelectrogens (including *Sphaerochaeta* [54,55], *Dechlorobacter* [56,57], *Arcobacter* [58,59], *Comamonas* [60,61], and *Hydrogenophaga* [62,63]), commonly existing in anode biofilm in BESs, significantly increased in EAB_25 LMH_ and EAB_50 LMH_ compared to EAB_0 LMH_. These putative exoelectrogens on EABs under filtration conditions might also improve electroactivity since most are equipped with redox-active components [54,55]. For instance, the *Sphaerochaeta* species has been found to participate in direct interspecies electron transfer and was capable of reducing the soluble Fe(III) oxides to Fe(II) [54,55]. *Lentimicrobium* [64], *Aquaspirillum* [9], and *Macellibacteroides* [65], reported to participate in EPS generation in BESs, were also enriched in EAB_25 LMH_ and EAB_50 LMH_, consistent with the above results. The results demonstrated that integrating electrical stimulation and filtration during EAB formation might motivate the growth of more electrogenic bacteria. To quantify the total amount of various electrogenic bacteria, the index was defined as equal to the biomass multiplied by the abundance of the electrogenic bacteria community (Table 1). The electrogenic bacteria index was consistent with the EPS protein content of EABs, showing that membrane filtration might promote the interaction between various electrogenic bacteria. Furthermore, it can establish a community that is more positive to producing electroactive substances, such as EPS proteins, to construct EABs with better performance.

**Table 1:** Comparison of electrogenic bacteria index under various fluxes.

Taxonomy	Relative abundance (%)			Reference
	EAB_0 LMH_	EAB_25 LMH_	EAB_50 LMH_	
*Geobacter*	47.6	0.3	0.1	[4]
*Sphaerochaeta*	1.3	2.7	9.6	[54,55]
*Dechlorobacter*	0.6	2.6	2.9	[56,57]
*Arcobacter*	0.3	1.2	0.6	[58,59]
*Comamonas*	0.2	1.3	0.5	[60,61]
*Hydrogenophaga*	0.3	1.1	0.4	[62,63]
Total abundance of the putative EAB	50.3	9.2	14.1	-
Biomass (μg cm^−2^)	8.6	79.2	106.6	-
Electrogenic bacteria index	4.3	7.3	15.0	-

### 3.5 Long-term operation stability

**Fig. 7:**
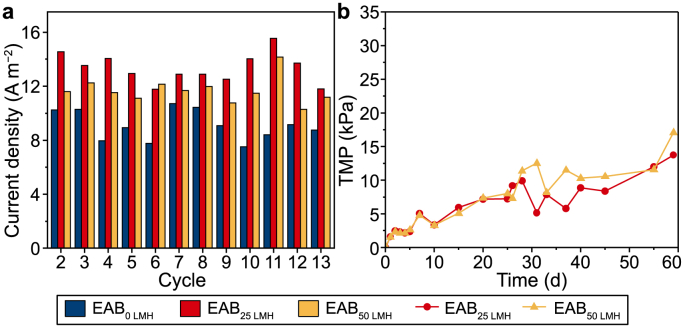
**a**, Maximum current density of three EABs in each cycle. **b**, TMP of EAB_25 LMH_ and EAB_50 LMH_ during the long-term operation.

Sustained and stable current production by EABs is a key element for the longevity and successful applications of BES systems [46]. Therefore, the long-term performance (60 days, 13 cycles) of three EABs was monitored to examine the roles of filtration on BES systems after the first cycle. The time-current profile is recorded in Fig. S5 and the maximum current density of three EABs in each cycle is depicted in Fig. 7a. The current density of three EABs during long-term operation exhibited the similar tendency with the first cycle, with the average current density of EAB_25 LMH_ (13.4 ± 1.1 A m^−2^) > EAB_50 LMH_ (11.6 ± 0.9 A m^−2^) > EAB_0 LMH_ (9.1 ± 1.1 A m^−2^). Transmembrane pressure (TMP) is a key parameter to evaluate the stability of the membrane system [66]. During the 60-d operation, both EAB_25 LMH_ (0.23 kPa d^−1^) and EAB_50 LMH_ (0.29 kPa d^−1^) exhibited a relatively low TMP-increasing rate, which was still in the TMP low-increase stage [67]. The low TMP-increasing rate in both EABs with filtration might be due to the enhanced anode oxidation. According to previous literature, the enriched exoelectrogens on the electrode would alleviate membrane fouling by anode oxidation, which leads to a dynamic equilibrium between fouling increase and decomposition, thus maintaining the operation stable [68]. The separation performance of EAB_25 LMH_ and EAB_50 LMH_ after 60-d operation is presented in Fig. S6. The matured EAB_25 LMH_ and EAB_50 LMH_ exhibited efficient separation performance for particulates and colloidal matters, with the effluent turbidity decreased by ∼94% compared to the domestic wastewater. The sustained current generation during long-term operation demonstrated the stability of EAB in this condition, and the robust EABs were demonstrated to act as a dynamic membrane for efficient sloid-liquid separation. Hence, the novel electroactive membrane would be capable of integrating into an anaerobic membrane bioreactor to replace the high-cost membrane in the future, in which the enhanced electron transfer by the EAB under filtration might improve biogas recovery and upgrade biogas [27,69]. Despite that, since EAB could be selectively enriched for some specific pollutants [12,20,53], these dynamic membranes might serve as an electroactive biocatalyst membrane for treating some pollutants, which might extend the use of BES in the future.

## 4 Conclusion

In this work, the integration of the filtration process into BESs was developed to accelerate EAB formation and enhance electrochemical performance. Results revealed that the dual functions of enhancing mass transfer and interception induced by filtration could significantly shorten *t*_start-up_ and enhance the electroactivity of EABs. Electrochemical property analysis also showed that the enhanced electroactivity of EABs under filtration conditions should be due to the increasing amount of redox-active components and the decreasing charge transfer resistance. Further studies demonstrated that filtration increased the secretion of microbial productions, modulated EPS compositions, and increased the stored electroactive substances. Moreover, the spatial distribution of viability and FISH analysis indicated that increasing biomass and exoelectrogens played crucial roles in enhancing electroactivity. The stability during long-term operation and efficient solid-liquid separation performance indicated that the novel electroactive membrane could be an alternative in membrane-based systems, likely extending the use of bioelectrochemical systems in wastewater treatment and energy recovery.

## CRediT authorship contribution statement

**Jinning Wang:** Methodology, Writing - Original Draft, Visualization, Formal Analysis. **Mei Chen:** Investigation, Conceptualization, Supervision, Funding Acquisition, Writing - Reviewing & Editing, Project Administration. **Jiayao Zhang:** Investigation. **Xinyi Sun:** Methodology, Data Curation. **Nan Li:** Conceptualization, Writing - Reviewing & Editing. **Xin Wang:** Conceptualization, Supervision, Funding Acquisition, Writing - Reviewing & Editing, Project Administration, Resources.

## Declaration of competing interest

The authors declare that they have no known competing financial interests or personal relationships that could have appeared to influence the work reported in this paper.
