# Supplementary material for: Dynamic membrane filtration accelerates electroactive biofilms in bioelectrochemical systems
Source: Environ Sci Ecotechnol. 2023 Dec 27;20:100375. doi: 10.1016/j.ese.2023.100375 (PMC10821169; doi:10.1016/j.ese.2023.100375)
Supplement: Multimedia component 1 [file mmc1.docx]

**Supporting information**

**Dynamic Membrane Filtration Accelerates Electroactive Biofilms in Bioelectrochemical Systems**

Jinning Wang^1^, Mei Chen^1,^*, Jiayao Zhang^1^, Xinyi Sun^1^, Nan Li^2^, Xin Wang^1,^*

*1. MOE Key Laboratory of Pollution Processes and Environmental Criteria/Tianjin Key Laboratory of Environmental Remediation and Pollution Control/College of Environmental Science and Engineering, Nankai University, No. 38 Tongyan Road, Jinnan District, Tianjin, 300350, China*

*2. School of Environmental Science and Engineering, Tianjin University, No. 35 Yaguan Road, Jinnan District, Tianjin 300350, China*

***Corresponding Author*.* E-mail: [xinwang1@nankai.edu.cn](mailto:xinwang1@nankai.edu.cn) (X. Wang); [meichen1228@nankai.edu.cn](mailto:meichen1228@nankai.edu.cn) (M. Chen);

**Contents**

**Sections**

**Section S1**. Electrochemical analysis of electroactive biofilm.

**Section S2.** The process of preparation for FESEM.

**Section S3.** Biomass and EPS analysis

**Section S4.** Procedure for the separation performance of EAB modules.

**Figures**

**Fig. S1.** Schematic representation of (a) the fabrication of conductive filtration module as DM substrate and (b) experimental set-up.

**Fig. S2.** SEM images of (a) pristine carbon cloth basement, (b) EAB_0 LMH_, (c) EAB_25 LMH_, and (d) EAB_50 LMH_.

**Fig. S3.** Electrochemical properties of three EABs in replicate reactors at first cycle stage. (a) Turnover cyclic voltammetry, (b) derivate cyclic voltammetry, (c) nonturnover cyclic voltammetry and (d) electrochemical impedance spectroscopy.

**Fig. S4.** The equivalent circuit of EABs with different transmembrane fluxes.

**Fig. S5.** Current output over time of three EABs.

**Fig. S6.** Turbidity removal by the matured EAB_25 LMH_ and EAB_50 LMH_.

**Tables**

**Table S1.** Composition and concentration of trace solution

**Table S2.** Composition and concentration of vitamin solution

**Table S3.** Comparison of EABs under various fluxes

**Table S4.** The α-diversity of inoculum and electroactive biofilms.

**Section S1**. Electrochemical analysis of electroactive biofilm.

Chronoamperometry (CA) was used to record the current every 100 s. When the highest current density was observed in the first cycle, turnover cyclic voltammetry (CV) was performed over a potential window ranging from −0.6 V to 0.2 V at a scan rate of 1 mV/s (stabilization period of 300 s). The first derivative CVs (DCVs) were calculated using the central difference quotient to further compare the discrepancy in the peak height and midpoint potentials. The nonturnover CV was performed after refreshing the medium without acetate for two days after the first cycle within a potential window between -0.6 V and 0.2 V (vs Ag/AgCl) at a scan rate of 1 mV/s. Electrochemical impedance spectroscopy (EIS) was carried out at a frequency range from 100 kHz to 10 mHz at the open circuit potential with an amplitude of 10 mV after the first cycle.

**Section S2.** The process of preparation for FESEM.

EAB samples were taken from the modules and immediately rinsed with deionized (DI) water followed by soaking into 2.5% of glutaraldehyde at 4℃ for 8 h. Then the samples were washed with 50 mM PBS buffer three times and once in DI water. Thereafter, the samples were dehydrated by increasing concentration of ethanol (30%, 50%, 70%, and 100%) each for 5 min. Finally, the samples were dried at room temperature and sputtered with platinum (Pt) before visualizing with FESEM.

**Section S3.** Biomass and EPS analysis

Biofilm samples were completely scraped off by sterile knives and collected in aseptic centrifuge tubes for microbial product analysis. These obtained samples were resuspended in PBS (50 mM) sterilized and filtered with 0.22-μm nylon syringe filter for further analysis. The ultrasonication (227.5 W, 15 min) was conducted by an ultrasonic cell disruption system (MIULAB, Hangzhou, China) to obtain a total protein extract. Extracellular polymeric substances (EPS) of the biofilms was extracted using a modified cation exchange resin method (Yan et al., 2021). The bicinchoninic acid method was performed to detect the protein content, following the instructions of the protein quantification kit (E112-02, Vazyme, Nanjing, China). The phenol-sulfuric acid method was carried out to measure the content of polysaccharide (DuBois et al., 1956).

**Section S4.** Procedure for the separation performance of EAB modules.

Silicon dioxide (SiO_2_) particles (average pore size = 2 μm) was used as surrogate for colloidal material to characterize the separation performance of the matured EAB_25 LMH_ and EAB_50 LMH_. The turbidities of the samples collected from effluent were detected by a turbidimeter.


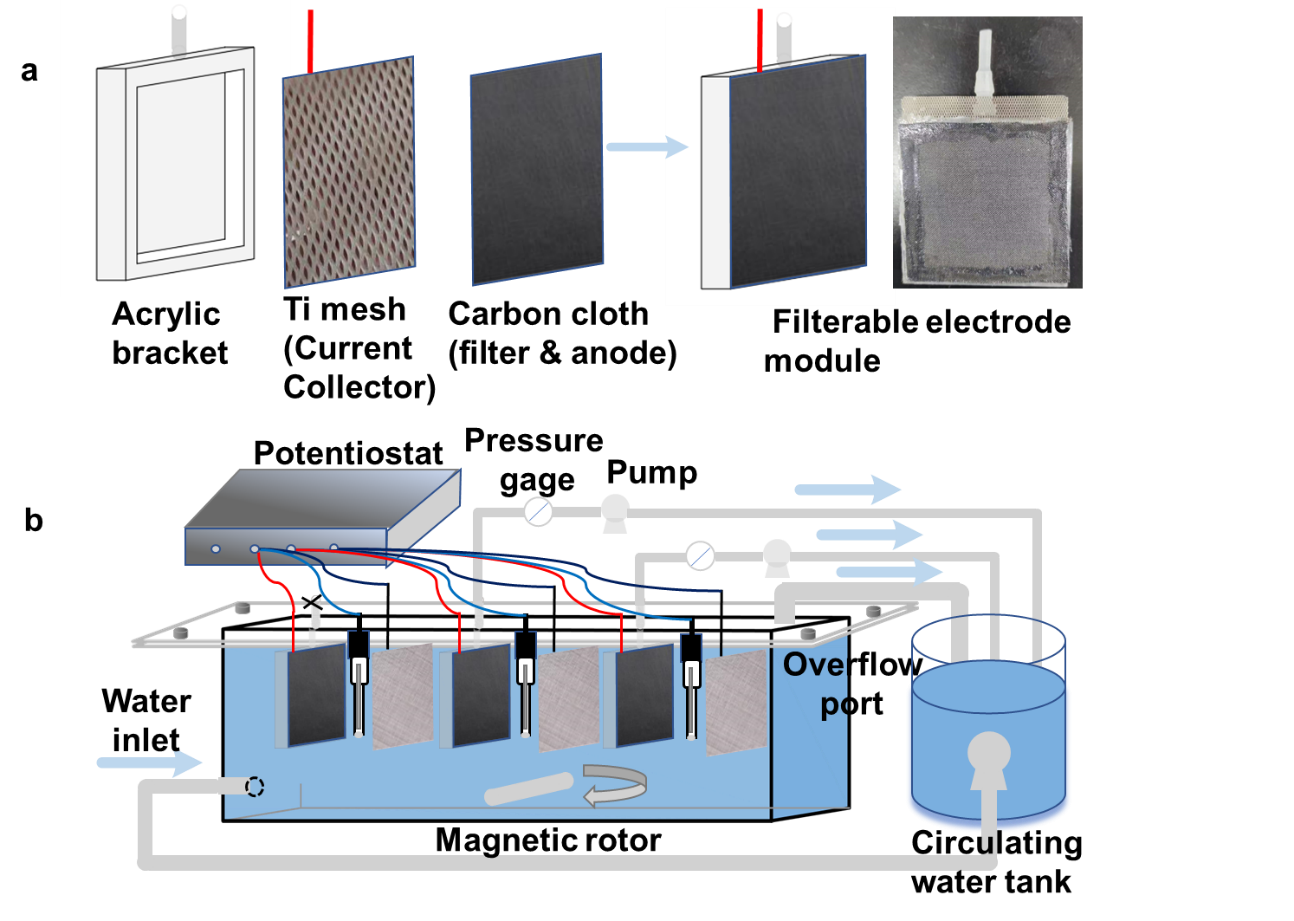


**Fig.S1.** Schematic representation of (a) the fabrication of conductive filtration module as DM substrate and (b) experimental set-up.


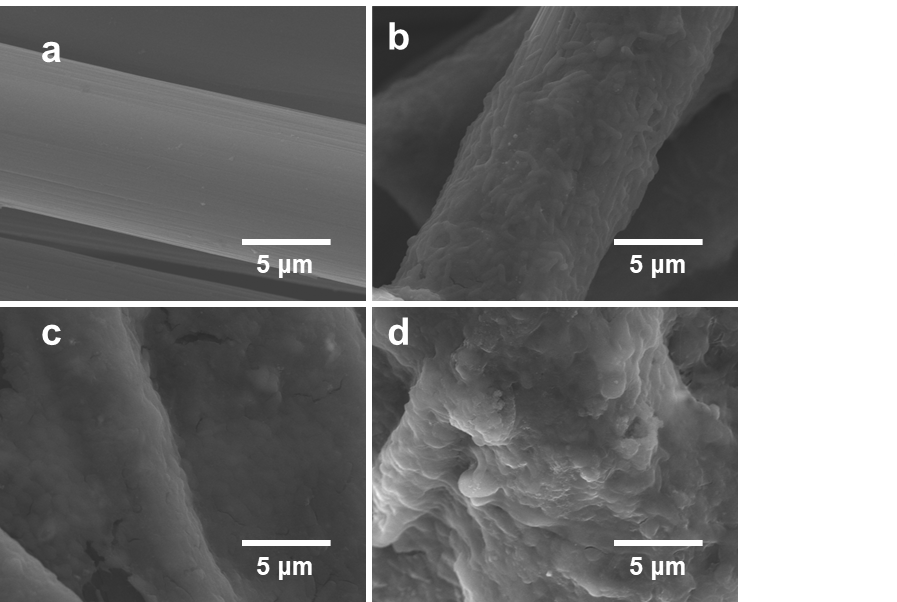


**Fig. S2.** SEM images of (a) pristine carbon cloth basement, (b) EAB_0 LMH_, (c) EAB_25 LMH_, and (d) EAB_50 LMH_.


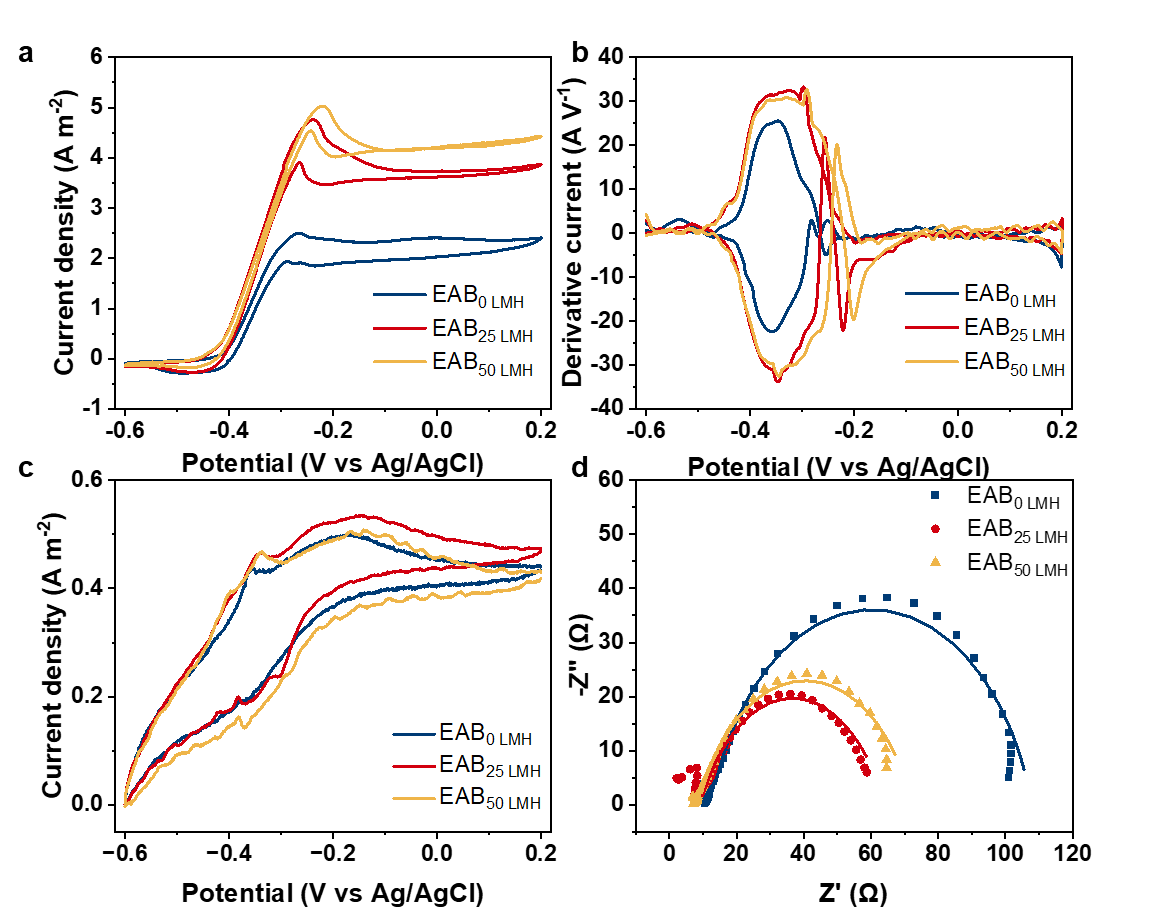


**Fig. S3.** Electrochemical properties of three EABs in replicate reactors at first cycle stage. (a) Turnover cyclic voltammetry, (b) derivate cyclic voltammetry, (c) nonturnover cyclic voltammetry and (d) electrochemical impedance spectroscopy.


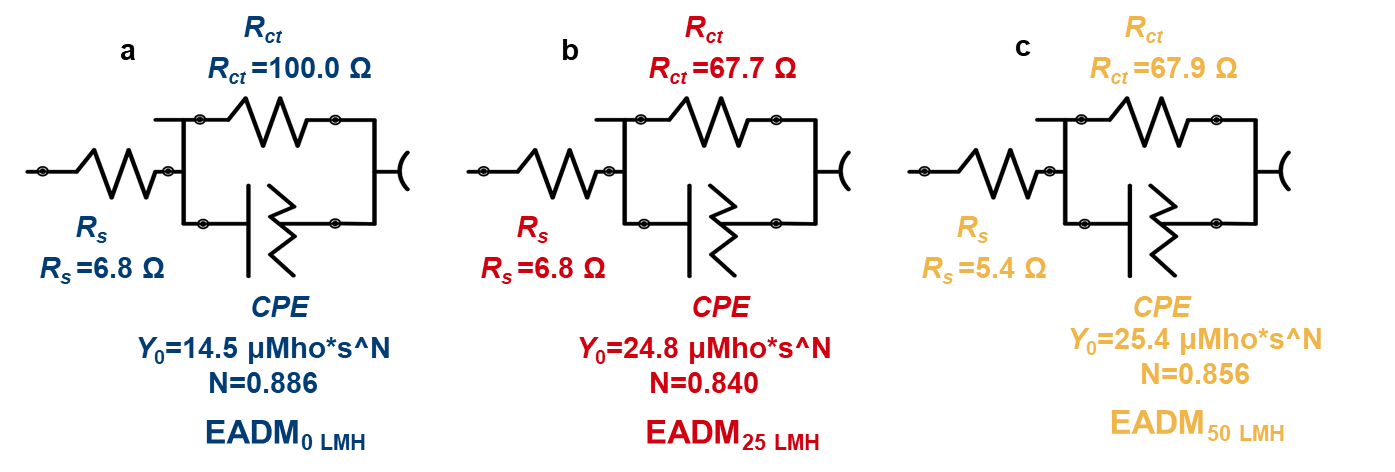


**Fig. S4.** The equivalent circuit of EABs with different transmembrane fluxes.

**Fig. S5.** Current output over time of three EABs.

**Fig. S6.** Turbidity removal by the matured EAB_25 LMH_ and EAB_50 LMH_.

**Table S1.** Composition and concentration of trace solution

| Reagent | Concentration (g/L) | Reagent | Concentration (g/L) |
| --- | --- | --- | --- |
| FeSO_4_ ·7H_2_O | 0.1 | MgSO_4_ | 3.0 |
| AlK(SO_4_) _2_ ·12H_2_O | 0.01 | CaCl_2_ ·2H_2_O | 0.1 |
| N(CH_2_COOH)_3_ | 2.0 | CuSO_4_ ·5H_2_O | 0.01 |
| NaCl | 1.0 | Na_2_WO_4_ ·2H_2_O | 0.025 |
| H_3_BO_3_ | 0.01 | ZnCl_2_ | 0.13 |
| MnSO_4_ | 0.5 | NiCl_2_ ·6H_2_O | 0.024 |
| Na_2_MoO_4_ | 0.025 | CoCl_2_ ·6H_2_O | 0.1 |

**Table S2.** Composition and concentration of vitamin solution

| Reagent | Concentration (g/L) | Reagent | Concentration (g/L) |
| --- | --- | --- | --- |
| Riboflavin | 5.0 | Vitamin B12 | 0.1 |
| Pantothenic acid | 5.0 | Niacinamide | 5.0 |
| Biotin | 2.0 | Vitamin b6 | 10.0 |
| P-Aminobenzoic acid | 5.0 | Lipoic acid | 5.0 |
| Ammonium sulphate | 5.0 | Folate | 2.0 |

**Table S3.** Comparison of EABs under various fluxes

| Sample | Coverage (%) | Biomass (μg/cm^2^) |
| --- | --- | --- |
| EAB_0 LMH_ | 19.4 ± 3.34 | 23.3 |
| EAB_25 LMH_ | 62.7 ± 8.7 | 75.2 |
| EAB_50 LMH_ | 80.3 ± 6.9 | 96.3 |

**Table S4.** The α-diversity of inoculum and electroactive biofilms

| Sample\Estimators | sobs | shannon | ace | chao | coverage |
| --- | --- | --- | --- | --- | --- |
| Inoculum | 1301 | 5.167333 | 1346.767715 | 1329.21393 | 0.998173 |
| EAB_0 LMH_ | 833 | 3.55095 | 1044.634193 | 1024.162393 | 0.996942 |
| EAB_25 LMH_ | 797 | 4.631526 | 978.028275 | 994.307692 | 0.997428 |
| EAB_50 LMH_ | 948 | 4.702555 | 1125.243997 | 1112.960938 | 0.996988 |
